# Experimental and Computational Study of MoS_2_-CeO_2_@MXene Hybrid Material for Energy Storage Applications

**DOI:** 10.3390/molecules31183321

**Published:** 2026-09-19

**Authors:** Ali Riza, Imran Murtaza, Syed Irfan, Sarfraz Ahmad, Rajab Hussain, Shafaat Hussain, Fayyaz Hussain, S. AlFaify

**Affiliations:** 1 Flexible Electronics Laboratory (FEL), Department of Physics, International Islamic University, Islamabad 44000, Pakistan; 2 Center for Advanced Electronics & Photovoltaic Engineering (CAEPE), International Islamic University, Islamabad 44000, Pakistan; 3 School of Intelligent Construction and Manufacturing, Sichuan Technology and Business University, Chengdu 611745, China; 4 School of Energy Chemical Engineering and Materials, Sichuan Technology and Business University, Meishan 620000, China; 5 Institute of Physics, The Islamia University of Bahawalpur, Bahawalpur 63100, Pakistan; 6 School of Chemical and Material Engineering, National University of Science and Technology, Islamabad 44000, Pakistan; 7 State Key Laboratory of Chips and Systems for Advanced Light Field Display, Beijing Key Lab of Nanophotonics and Ultrafine Optoelectronic Systems, School of Physics, Beijing Institute of Technology, Beijing 100081, China; 8 Materials Simulation Research Laboratory (MSRL), Institute of Physics, Bahauddin Zakariya University, Multan 60800, Pakistan; 9 Advanced Functional Materials & Optoelectronics Laboratory (AFMOL), Department of Physics, Faculty of Science, King Khalid University, Abha 61421, Saudi Arabia

**Keywords:** Ti_3_C_2_Tx, MoS_2_, CeO_2_, ternary composite, Density Functional Theory (DFT), supercapacitor, energy storage

## Abstract

The paper includes the synthesis and characterization of a new ternary composite electrode material based on MXene (Ti_3_C_2_T_x_), Molybdenum Disulfide (MoS_2_), and Cerium Oxide (CeO_2_) nanoparticles for supercapacitor applications. The heterostructure was prepared by a hydrothermal technique with a 1:1:1 ratio, ensuring a well-integrated heterostructure. The successful anchoring and uniform distribution of MoS_2_ and CeO_2_ on the layered MXene matrix were confirmed by structural and morphological analysis using X-ray diffraction (XRD) and scanning electron microscopy (SEM). Electrochemical performance measurements such as cyclic voltammetry (CV), galvanostatic charge–discharge (GCD), and electrochemical impedance spectroscopy (EIS) were used to collectively show that the composite has a higher specific capacitance of 1220 F g^−1^ at 1 A g^−1^, good cycling stability of 90, and content retention at 5K cycles. A negative binding energy, calculated using Complementary Density Functional Theory (DFT), shows strong interfacial interaction and the structural stability of the composite. Moreover, the analysis of the Density of States (DOS) showed indicators of metallic-like behavior due to the interaction of Ti-d, Mo-d, and Ce-f orbitals, which largely reduces the energy barrier to the flow of electrons. These findings demonstrate the synergistic nature of high conductivity by MXene, pseudocapacitance by the metal oxides/sulfides, and improved redox performance, and thus make the MoS_2_-CeO_2_ @ MXene hybrid an attractive target in the next generation of energy storage devices.

## 1. Introduction

It has been observed that fossil fuels are being depleted at an alarming rate, and increased demand for sustainable forms of energy has led to the development of high-performance energy storage systems that are efficient and meet high standards. Although traditional batteries have high energy density, they have been known to have low charging rates and short cycle lives [1,2,3,4]. Electrochemical capacitors, or supercapacitors (SCs), have become a plausible alternative, covering the gap between traditional capacitors and batteries. They offer high power density, high charge–discharge rate, and outstanding cycle stability, thus making them indispensable in the design of both portable electronics and electric vehicles [5,6]. Nonetheless, a critical bottleneck to the widespread implementation of SCs is the comparatively low energy densities of commercial electrode materials (i.e., activated carbon), which limit their operation time. As a result, recent studies aim to design new electrode substances that possess high conduction capability and a high redox potential to enhance specific capacitance without interfering with stability [7,8]. Attention to 2D (two-dimensional) materials received a groundbreaking boost with the discovery of graphene, mostly because of the combination of a large surface-to-volume ratio and controllable electronic conductivity. The most notable materials are MXenes, a new group of 2D transition metal carbides/nitrides, that have attracted considerable attention since their discovery in 2011.MXenes are so-called etching products obtained by etching the A layer (usually Al) of layered MAX phases, giving them a special combination of metallic electrical conductivity and hydrophilia [9,10,11]. The most studied MXene is Ti_3_C_2_T_x_ (x = surface functional groups -OH, -O, -F), which allows the general formula to have a versatile surface chemistry. Despite these strengths, MXene nanosheets are affected by serious restacking due to van der Waals forces, which significantly decreases the accessible surface area and impedes ion transport [12,13,14,15]. To resolve the problem of restacking and increase capacitance, it is common to hybridize MXenes with pseudocapacitive materials, including transition metal dichalcogenides (TMDs). A typical TMD is molybdenum disulfide (MoS_2_), which is known to have a high theoretical capacity and a layered structure, making it efficient for ion intercalation. However, it is inherently poor in electrical conductivity and structural stability (volume expansion), so pure MoS_2_ is impractical as a cycling medium. Thus, by developing heterostructures in which conductive MXene nanosheets can be used as a substrate on which MoS_2_ is grown, the restacking of MXene can be avoided, and the increase in MoS_2_ conductivity can be achieved at the same time [16,17,18,19,20]. To achieve higher electrochemical performance, the introduction of rare earth metal oxides is an effective strategic additive. Cerium Oxide (CeO_2_) stands out as one of the fastest redoxers with the ability to switch between the states of Ce^3+^ and Ce^4+^ as well as having high oxygen vacancies. These voids serve as active centers of charge storage and also promote quick diffusion of ions. Moreover, the CeO_2_ crystal structure provides good stability, which could reduce the mechanical decay of the electrode in long-term cycling [21,22,23,24]. This research describes an effortless hydrothermal method to produce a new ternary hybrid, MoS_2_-CeO_2_@MXene. This design exploits a synergistic mechanism where: (1) MXene offers a highly conductive 2D structure; (2) MoS_2_ offers a high pseudocapacitance; and (3) CeO_2_ nanoparticles open oxygen vacancies and inhibit agglomeration between nanosheets. Although the pristine MoS_2_ (particularly in its standard (2H) phase) acts as a semiconductor and suffers from poor intrinsic electrical conductivity, which leads to limited individual electrochemical rate capability, MoS_2_/MXene composites have demonstrated considerable promise as supercapacitor electrodes. However, challenges associated with the intrinsically limited conductivity of MoS_2_ and the restacking tendency of both MoS_2_ and MXene continue to hinder their electrochemical performance. To address these limitations, the incorporation of a redox-active metal oxide represents an effective strategy for introducing additional electrochemically active sites while simultaneously enhancing interfacial charge transfer characteristics. Among the various metal oxides, CeO_2_ is particularly attractive because of its reversible Ce^3+^/Ce^4+^ redox couple and defect-rich surface chemistry, both of which can contribute to enhanced electrochemical activity. Therefore, integrating CeO_2_ into an MoS_2_/MXene framework is expected to generate synergistic electrochemical effects, where MXene serves as a highly conductive electron transport pathway, MoS_2_ provides pseudocapacitive charge storage capability, and CeO_2_ supplies additional faradaic active sites. Furthermore, the resulting ternary heterostructure can create multiple interconnected interfaces, improve active-site exposure and electrolyte accessibility, and accelerate charge transfer kinetics. While binary MoS_2_/MXene and metal oxide/MXene composites have been extensively investigated for energy storage applications, the specific synergistic contribution of CeO_2_ within an MoS_2_/MXene ternary architecture remains largely unexplored. Consequently, the rational design and systematic investigation of a CeO_2_/MoS_2_/MXene electrode for supercapacitor applications constitute the primary novelty and scientific significance of the present study [25,26]. Recent studies have demonstrated that the electrochemical performance of MXene-based supercapacitors can be significantly enhanced through interface engineering and heterostructure design, which promote charge transfer kinetics and ion accessibility. The growing demand for sustainable energy technologies has encouraged the development of advanced electrode materials with high conductivity, abundant active sites, and rapid charge transfer kinetics [27]. In addition, oxygen vacancy engineering has emerged as an effective strategy to regulate the electronic structure, generate additional redox-active sites, and improve ion/electron transport, thereby enhancing capacitive performance [28]. Our synthesized composite shows a very high specific capacitance of 1220 F g^−1^ at 1 A g^−1^, similar to most previously published MXene-based systems. Moreover, we use Density Functional Theory (DFT) calculations to study the electronic interactions at the interface, providing theoretical understanding of the stability and conductivity of the hybrid material.

## 2. Results and Discussion

### 2.1. XRD Analysis

**Figure 1:**
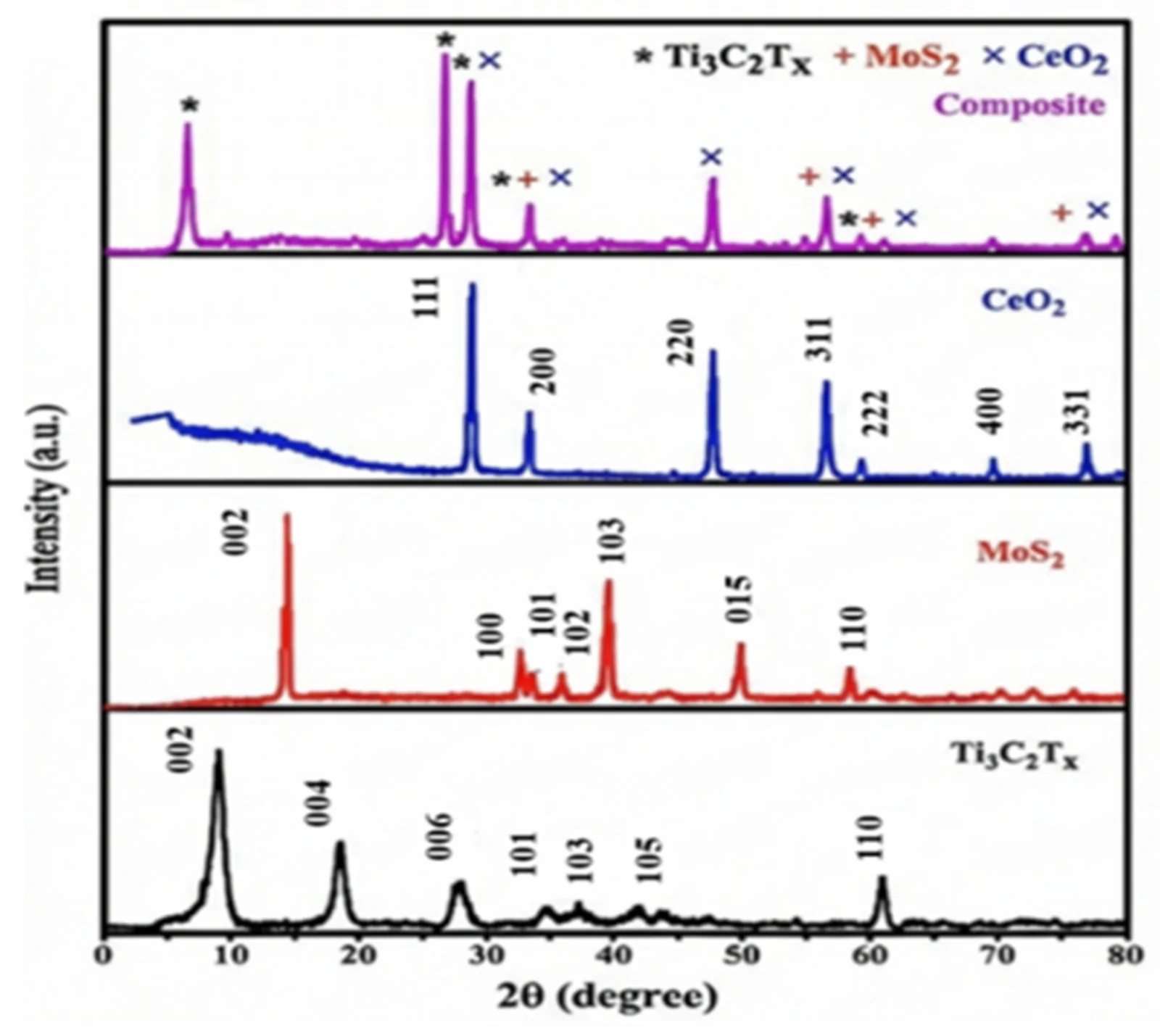
X-ray diffraction spectroscopy (XRD) patterns of Ti_3_C_2_T_x_, MoS_2_, CeO_2_, and the their composite (MCM).

Figure 1 demonstrates that X-ray diffraction (XRD) was used to study the crystallographic structure and purity of the phases of the synthesized materials. The XRD pattern of the pristine Ti_3_C_2_T_x_ MXene exhibits well-defined characteristic peaks indexed to the (002), (004), (006), (101), (103), (105), and (110) planes [25,26,27,28,29,30,31]. The sharp intensity of the (002) reflection confirms the successful selective etching of the MAX phase and the preservation of the layered MXene structure, which is essential for providing a conductive backbone. The MoS_2_ 2θ pattern displays a hexagonal crystal structure with distinct peaks at 14.48°, 32.80°, and 33.53°, corresponding to the (002), (100), and (101) planes, respectively [2,16,17,20,25]. Similarly, the CeO_2_ spectrum confirms a cubic fluorite structure (space group Fm-3m) with key diffraction peaks at 28.58° (111), 33.30° (200), and 47.65° (220) [23,26,32,33]. The diffraction peaks of hexagonal 2H-MoS_2_ and cubic fluorite CeO_2_ can be indexed using PDF/JCPDS cards 37–1492 and 34–0394, respectively. The parent Ti_3_AlC_2_ MAX phase can be compared with PDF/JCPDS card 52–0875. A single fixed JCPDS card should not be assigned uncritically to Ti_3_C_2_T_x_ because its basal reflections vary with surface terminations, hydration, intercalation, and interlayer spacing.

In the spectrum of the ternary MoS_2_-CeO_2_@MXene composite, diffraction features from all three constituents coexist, confirming the successful formation of the heterostructure. Notably, the intensity of the MoS_2_ (002) peak at ~14° is significantly suppressed in the composite compared to the pure material. This inhibition indicates that the MXene substrate is a good inhibitor of vertical restacking of MoS_2_ nanosheets. This structural optimization is directly related to improved electrochemical activity, as it prevents agglomeration and exposes a greater area of the structure to the adsorption of electrolyte ions, which is the main factor leading to the increased specific capacitance of 1220 F g^−1^ in this work. Moreover, some more area at 48° shows a peak shift and convolution between the CeO_2_ (~47°) and MoS_2_ (~49°) reflections. This shift, indicating lattice strain and close interfacial contact between the components, provides a low-resistance pathway for ion transfer, which explains the reduced charge transfer resistance (Rct) and improved rate capability [34,35].

### 2.2. FTIR Analysis

**Figure 2:**
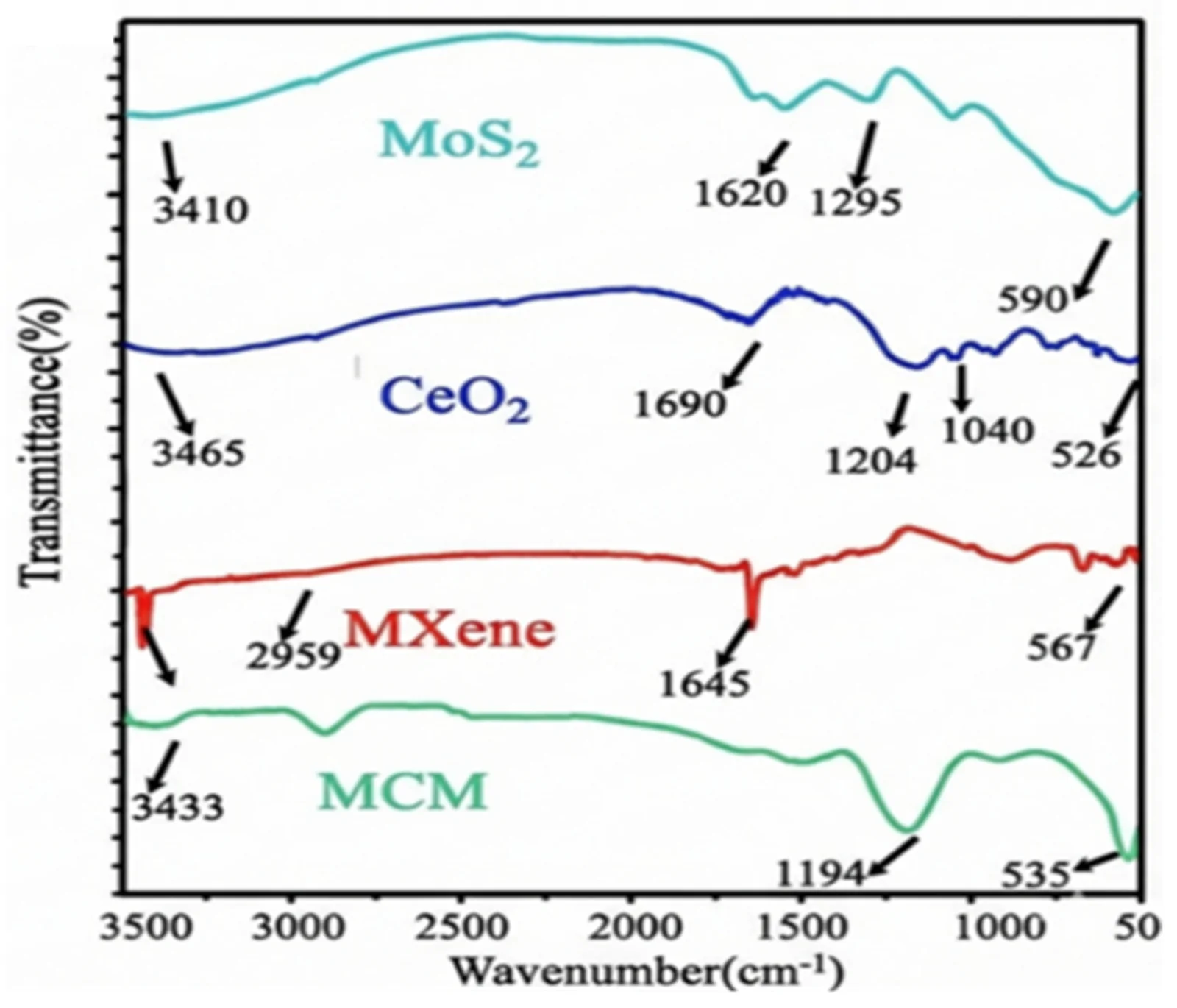
FTIR analysis of Ti_3_C_2_T_x_, MoS_2_, CeO_2_, and their composite (MCM).

Fourier Transform Infrared Spectroscopy (FTIR) as shown in Figure 2 was used to analyze the surface functional groups and chemical bonding of the samples. The FTIR spectra were recorded using the ATR mode. The pure MoS_2_ spectrum has a broad absorption around 3410 cm^−1^, suggesting that the O-H stretch of moisture adsorbed onto the sample is present in the spectrum, and Mo-S vibration is observed close to 590 cm^−1^. The strong stretching vibration of the Ce-O bond at 526 cm^−1^ predominates in the CeO_2_ spectrum, as well as surface hydroxyl groups. The MXene spectrum identifies the termination groups of the surface, such as C-H stretching at 2959 cm^−1^, O-H bending at 1645 cm^−1^, and Ti-O/Ti-C vibrations at 567 cm^−1^ [21,25,26,36,37]. The FTIR spectrum of the MCM composite indicates that the chemical environment is coherent and does not lead to the loss of the main characteristics of the individual constituents of the composite. Importantly, the presence of the metal–oxygen/metal–sulfur bands (approximately 535 cm^−1^ and 1194 cm^−1^) and a visible, shifted O-H band at 3433 cm^−1^ proves that MoS_2_ and CeO_2_ are chemically bound to the hydrophilic MXene surface. This strong chemical integration serves two purposes: first, the large number of hydrophilic functional groups increases the wettability of the electrode, which results in faster diffusion of electrolyte ions and allows the electrode to charge–discharge more quickly. Second, intense interfacial anchoring inhibits the detachment of active material during repeated volume expansion and contraction of cycling, which is the essential process of achieving exceptional cyclic stability (>90% retention) [26,38] of the composite.

### 2.3. SEM Analysis

**Figure 3:**
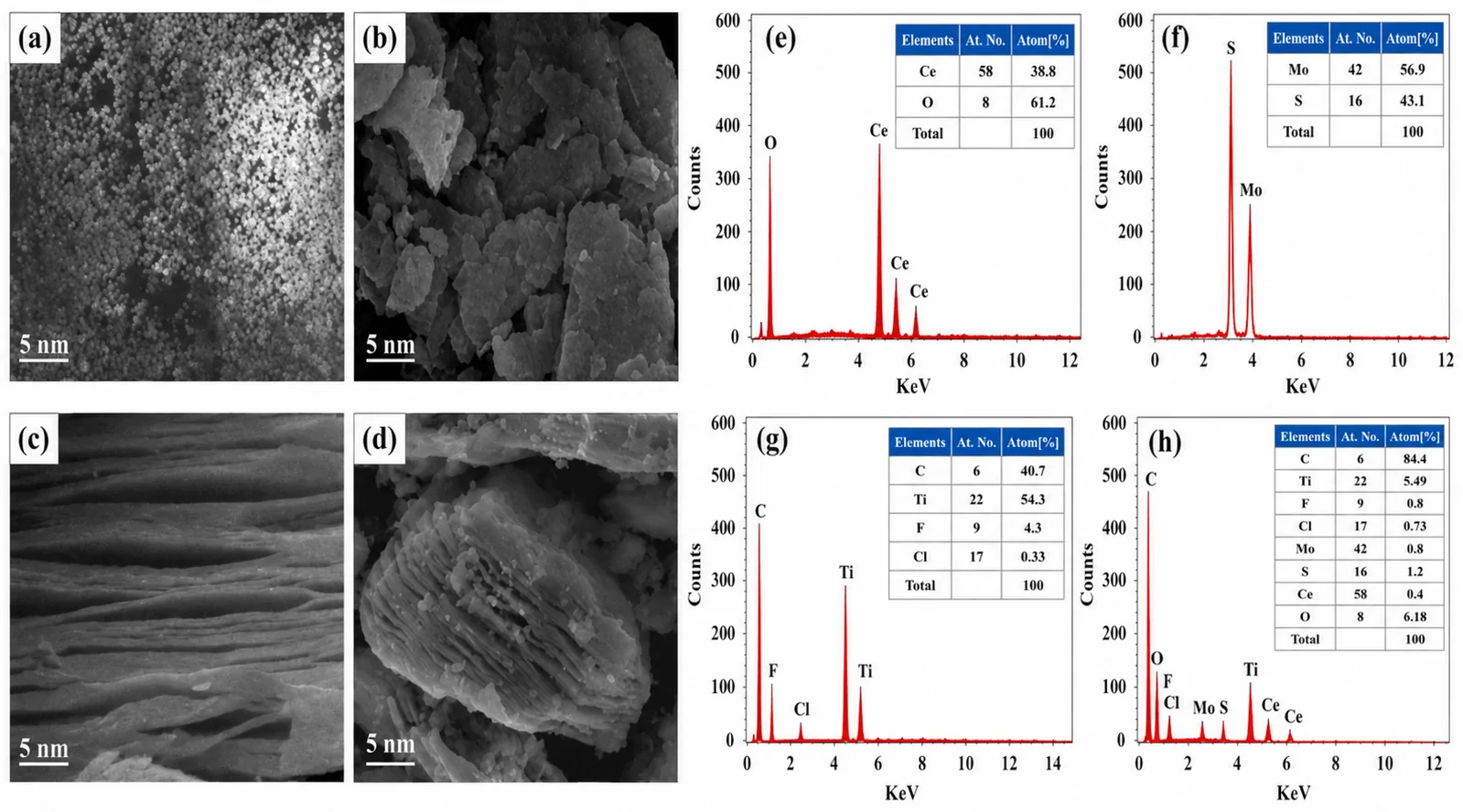
SEM images of (**a**) CeO_2_, (**b**) MoS_2_, (**c**) MXene, and (**d**) the MXene-based composite (MCM), along with the EDS spectra of (**e**) CeO_2_, (**f**) MoS_2_, (**g**) MXene, and (**h**) the MXene-based composite (MCM).

SEM was used to investigate the surface morphologies of individual materials and their composite as depicted in Figure 3. The morphology of CeO_2_ is presented in Figure 3a shows a nanoparticulate morphology, indicating the formation of fine CeO_2_ nanoparticles. whhle Figure 3b, MoS_2_ exhibits a layered/flake-like morphology. Conversely, Figure 3c shows that MXene has a layered, sheet-like structure that enables quick ionic transport and electronic conduction because of its two-dimensional structure and conductive surface. Notably, Figure 3d indicates that the hybrid structure is well integrated, with MoS_2_ and CeO_2_ nanoparticles evenly attached to the layered Ti_3_C_2_Tx material. This coupled morphology favors synergistic effects, as MXene can be combined with the high conductivity of MoS_2_ and CeO_2_ with pseudocapacitive properties. The scaffold employed is layered MXene, as it aids in the dispersion of the nanoparticles and prevents restacking, keeping the high surface area intact and, consequently, enhancing electrolyte accessibility to the nanoparticles. This hierarchical assembly is expected to achieve better electrochemical performance of the composite in practical supercapacitor applications, providing improved charge storage capacity, conductivity, and rate capability [18,26,32,33,37] of MXene. This feature facilitates rapid ion transport and electronic conductivity due to its two-dimensional architecture and conductive surface, resulting in improved electrochemical performance.

### 2.4. Raman Analysis

**Figure 4:**
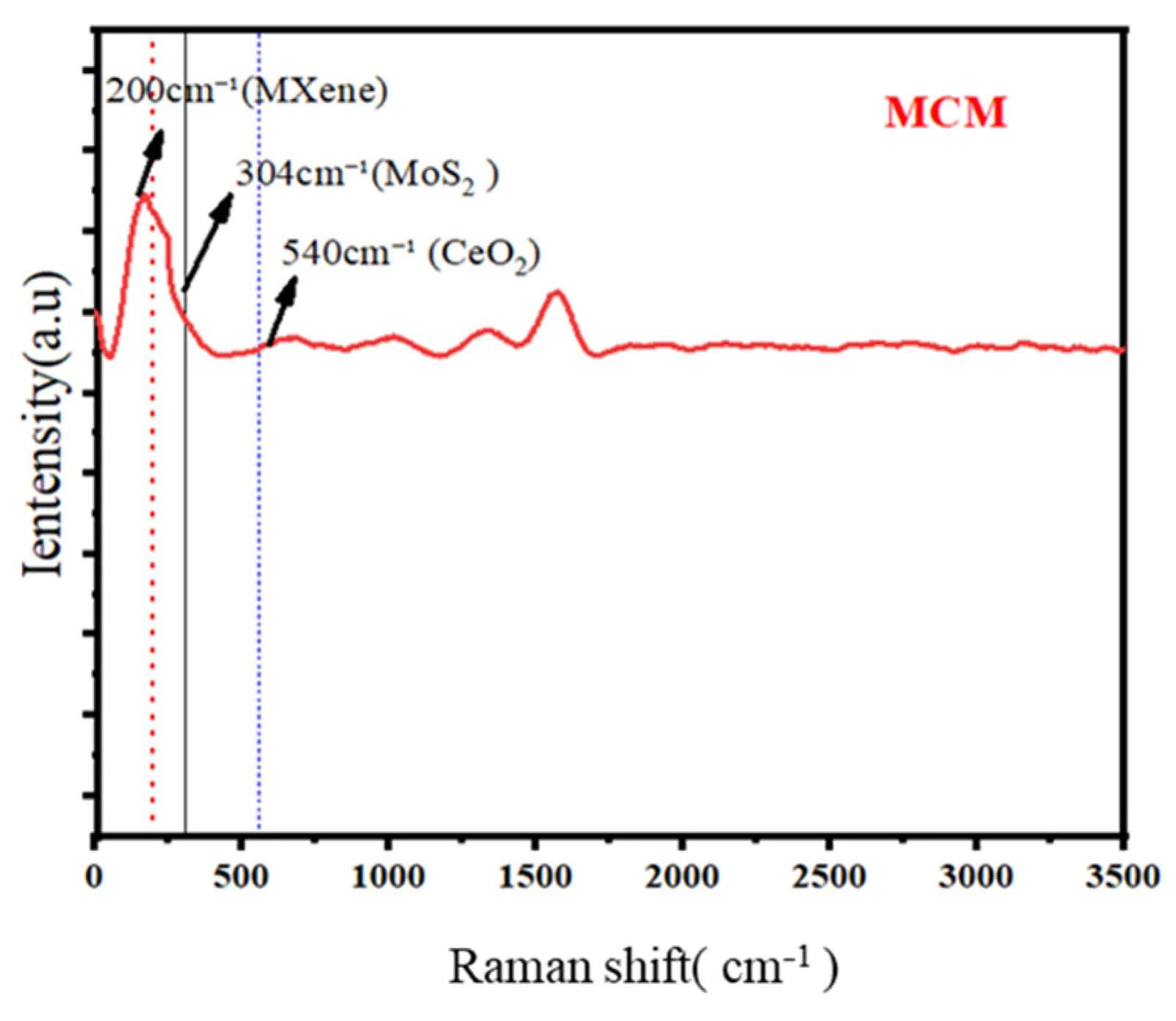
The Raman analysis of the composite (MCM).

The Raman spectrum of the MoS_2_/CeO_2_/MXene composite (MCM) as shown in Figure 4, exhibits the characteristic vibrational modes of all constituent materials. The peaks at approximately 304 cm^−1^ (E_2_g^−1^) correspond to the in-plane and out-of-plane vibrations of MoS_2_, while the intense band near 550 cm^−1^ is assigned to the F_2_g mode of fluorite-structured CeO_2_. Additionally, the broad bands observed in the 200 cm^−1^ region are attributed to Ti-C vibrations and surface functional groups of MXene (Ti_3_C_2_T_x_). The presence of these characteristic peaks, along with slight shifts and broadening, confirms the successful integration of MoS_2_, CeO_2_, and MXene and indicates strong interfacial interactions within the composite.

### 2.5. BET Analysis

**Figure 5:**
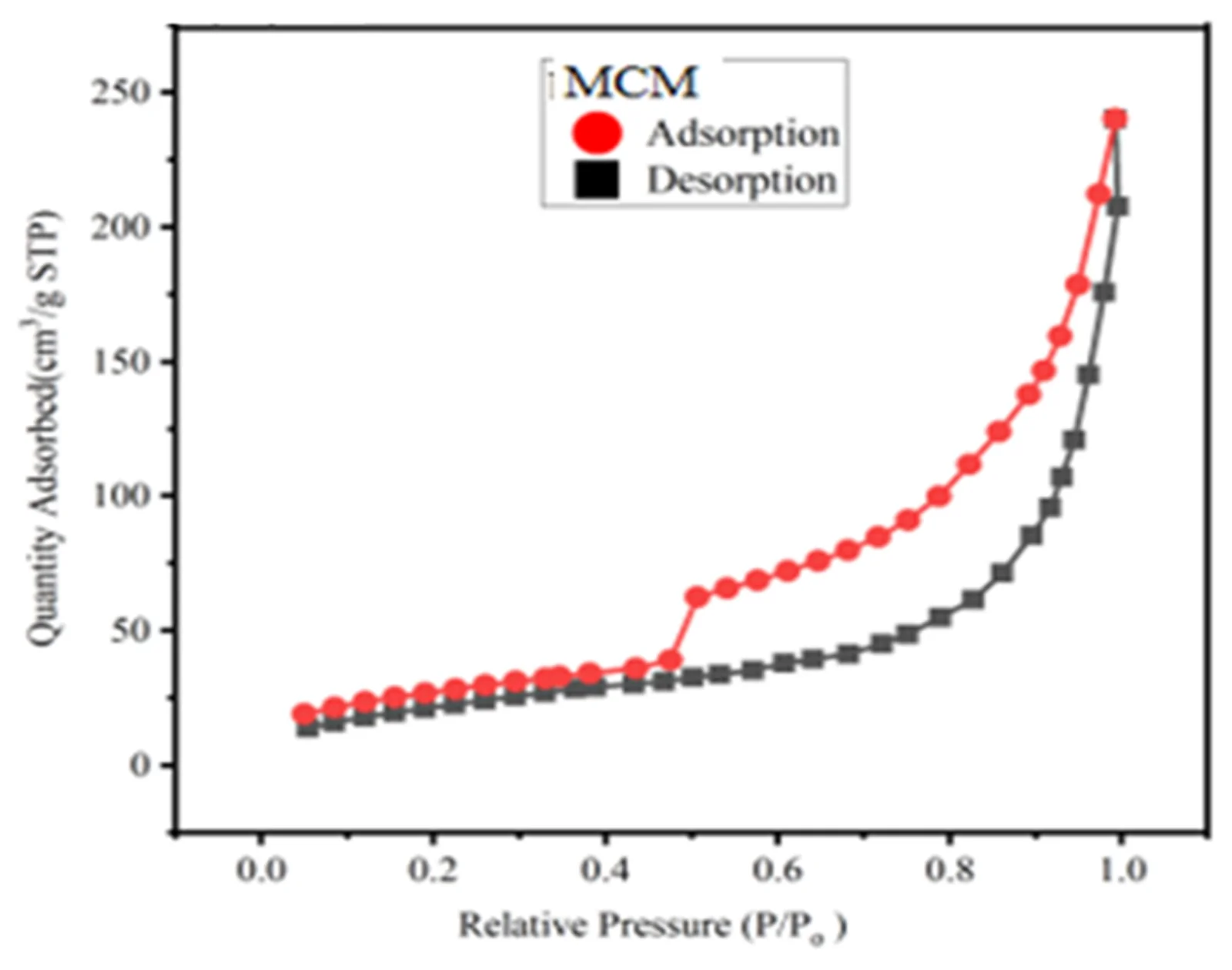
BET analysis of the composite (MCM).

The N_2_ adsorption–desorption isotherm of the MoS_2_/CeO_2_/MXene composite (MCM) as shown in Figure 5 exhibits a characteristic mesoporous behavior with a distinct adsorption step at P/P_0_ ≈ 0.5 and a broad hysteresis loop extending to high relative pressures. The nitrogen uptake increases from ~35 to ~65 cm^3^ g^−1^ STP in the capillary condensation region and reaches ~240 cm^3^ g^−1^ STP near P/P_0_ = 1.0, indicating a high pore volume and well-developed mesoporosity. Such a porous structure can facilitate electrolyte accessibility and rapid ion diffusion, making the composite suitable for high-performance supercapacitor electrodes.

## 3. Three-Electrode Analysis

### 3.1. Cyclic Voltammetry (CV) Study

**Figure 6:**
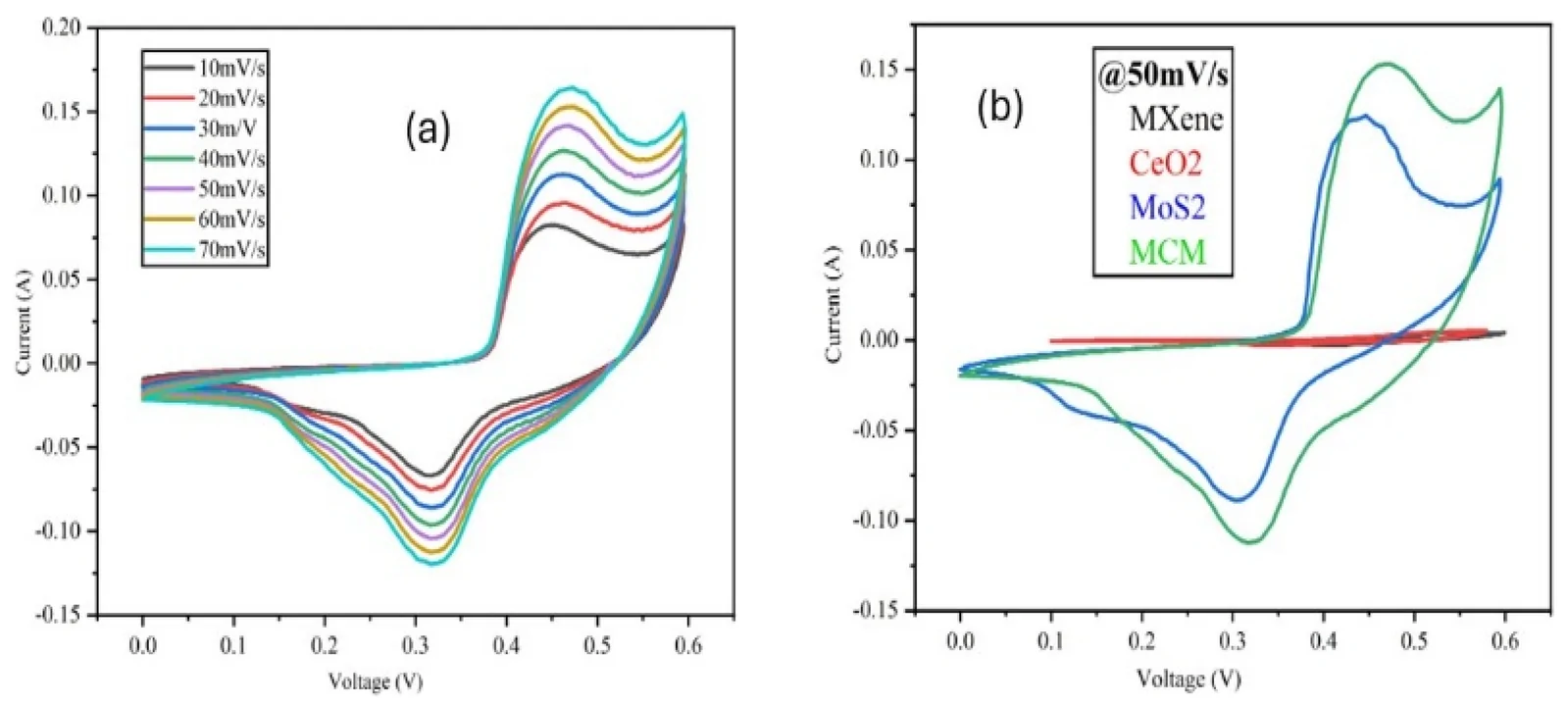
Cyclic voltammetry (CV) analysis of the samples: (**a**) CV analysis of the composite (MCM) at different scan rates; (**b**) CV analysis of MXene, MoS_2_, CeO_2_, and their composite (MCM) at same scan rate.

Figure 6 shows the cyclic voltammetry (CV) analysis of the prepared samples, which explains the charge storage rate and redox activity of the sample. The CV curves of the composite (MCM) at scan rates between 10 and 70 mV s^−1^ in panel (a) clearly show a pair of redox peaks, which is evidence of the presence of a pseudocapacitive process that is dominated by faradaic reactions. The current response increases with the scan rate, indicating efficient electrochemical kinetics and high-rate capability, whereas the stability of the curve shape indicates that even rapid scans can be run without losing the curves. In panel (b), the CV responses of individual materials (MXene, CeO_2_, and MoS_2_) and the composite (MCM) have been compared at a scan rate of 50 mV s^−1^. Notably, the pure CeO_2_ electrode exhibits a relatively featureless CV curve with a minimal current response and small integrated area, whereas MoS_2_ and MXene demonstrate the average features of pseudocapacitive materials. However, in comparison, the composite (MCM) has a much higher integrated area and redox features, which support the synergistic interaction of its components and better charge storage capacity, thus rendering it a potential electrode in supercapacitor applications [8,23,26,28,33,36].

### 3.2. Galvanostatic Charge–Discharge (GCD) Measurements

**Figure 7:**
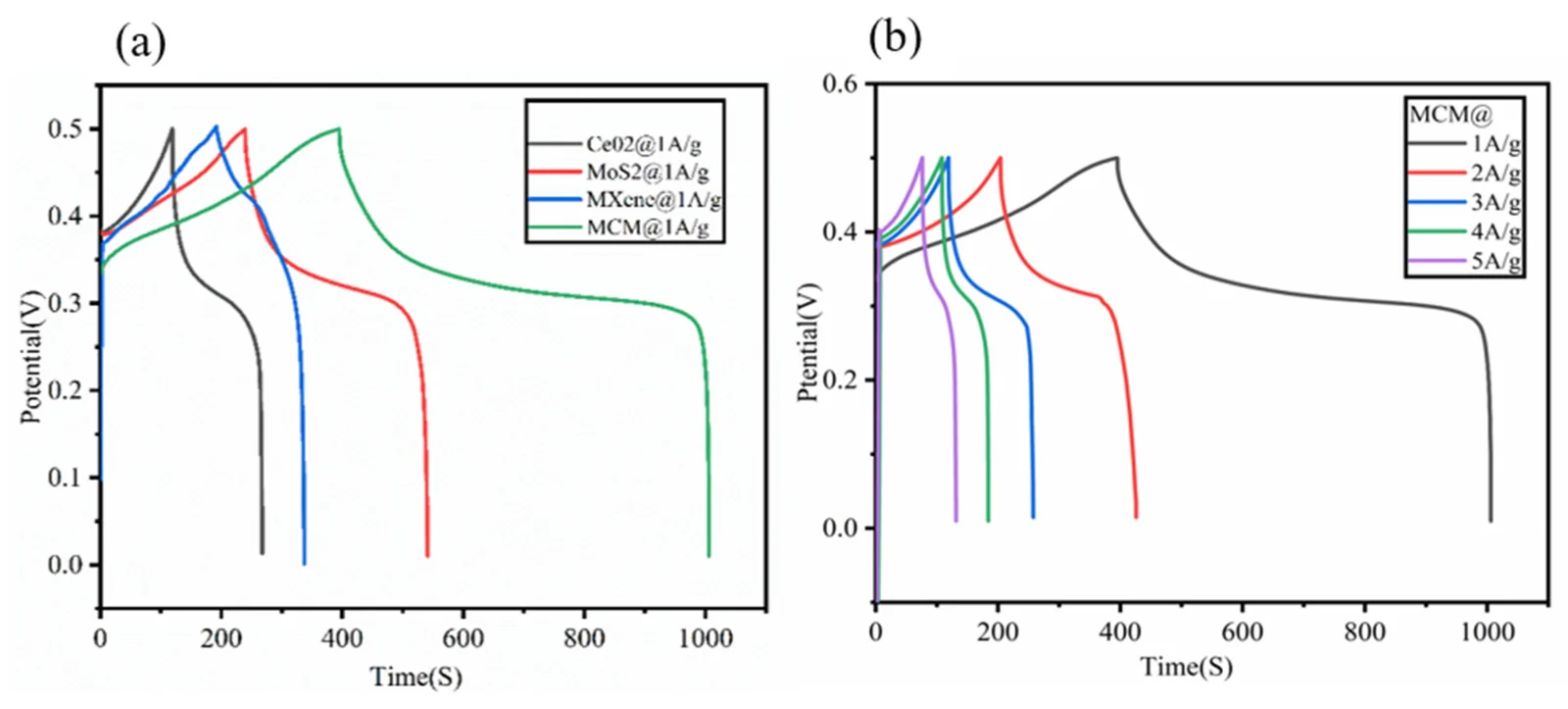
(**a**) Galvanostatic charge–discharge (GCD) curves of the individual components Ti_3_C_2_T_x_, MoS_2_, and CeO_2_ and the ternary composite (MCM) at a constant current density of 1 A g^−1^. (**b**) GCD profiles of the MCM composite at different current densities: 1, 2, 3, 4, and 5 A g^−1^.

**Figure 8:**
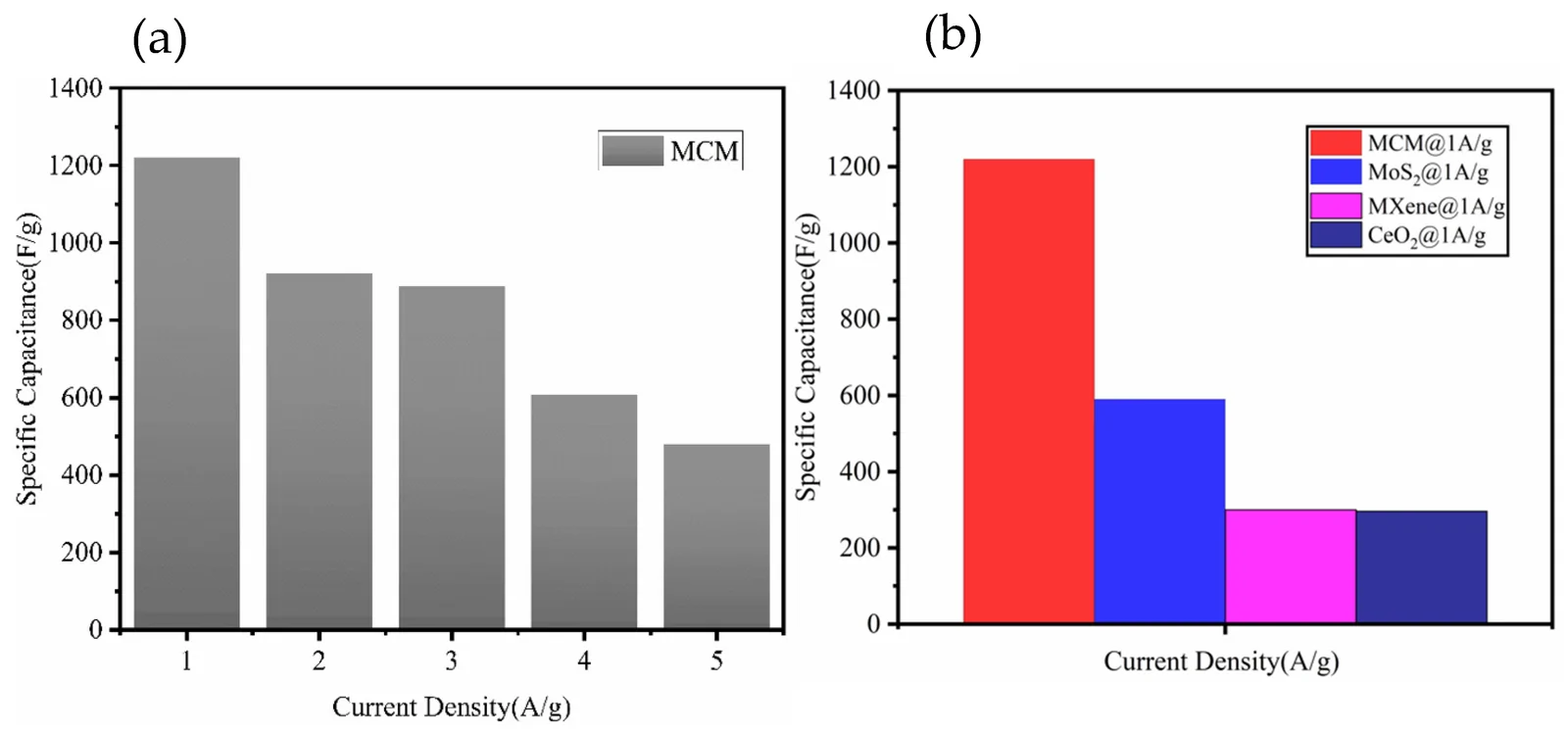
(**a**) Specific capacitance values for the MCM composite at different current densities (1–5 A g^−1^) and (**b**) for different samples at the same current density (1 A g^−1^).

**Figure 9:**
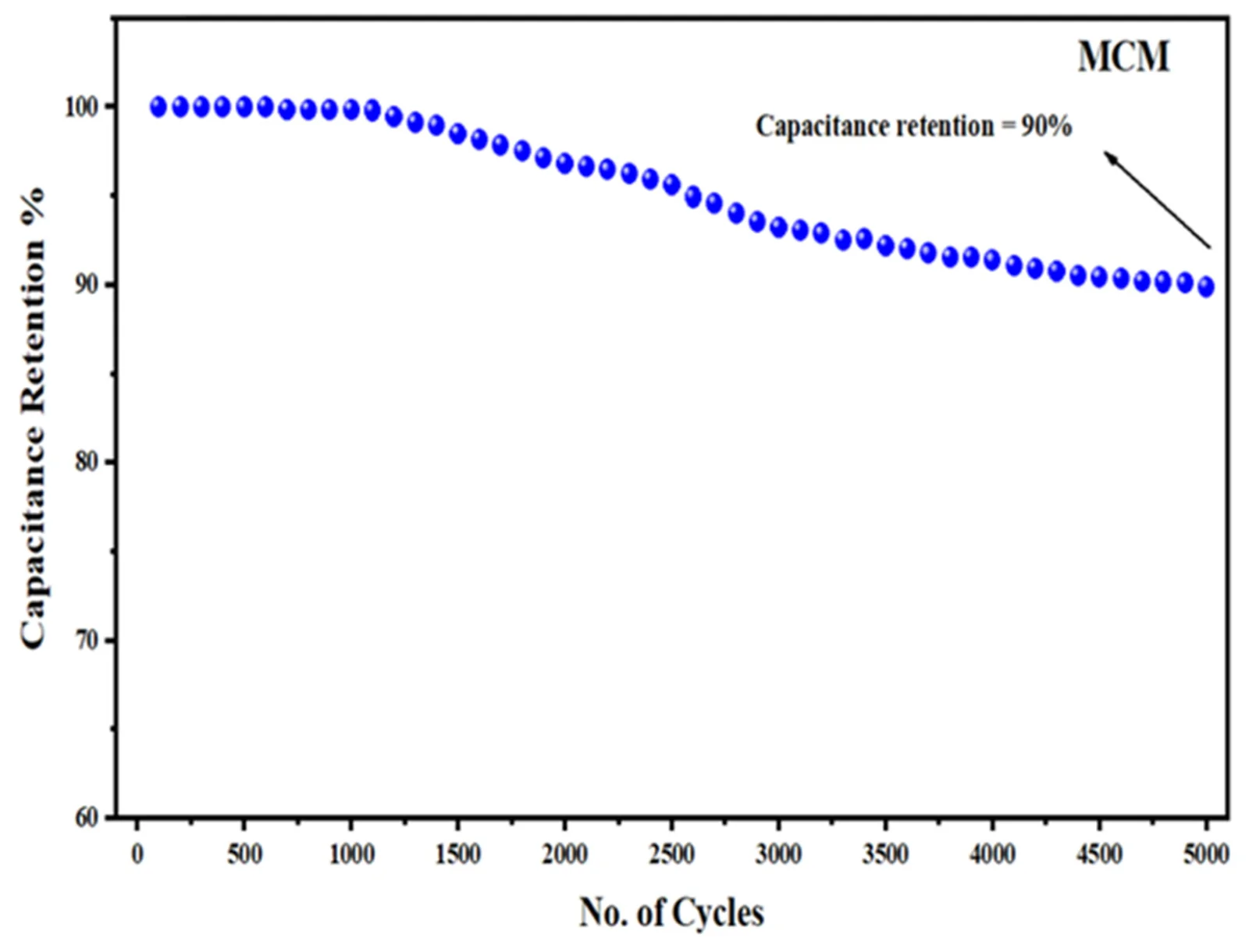
Stability of the MCM composite over 5000 cycles.

Figure 7a shows the galvanostatic charge–discharge (GCD) curves of the individual components Ti_3_C_2_T_x_, MoS_2_, and CeO_2_, and Figure 7b presents the ternary galvanostatic charge–discharge (GCD) curves of the composite (MCM) at a constant current density of 1 A g^−1^. CeO_2_ is the material with the shortest discharge time among the individual materials, which is a sign of low capacitive ability, which is mainly attributed to poor electronic conductivity. MXene and MoS_2_ show moderate performance, which is attributed to properties inherent to pseudocapacitance and conductance, respectively. On the other hand, the composite (MCM) provides the most prolonged discharge time and discrete plateau area, which indicates the synergistic influence of the three constituents. The combined architecture of the composite (MCM)is a successful synthesis that utilizes the high surface area of MoS_2_ and the redox-active properties of CeO_2_, as well as the outstanding conductivity and layered geometry of MXene, to provide improved charge storage capacity and ionic transport [23,25,28]. Herein, Figure 7b shows the GCD profiles of the composite (MCM) at different current densities (1, 2, 3, 4, and 5 A g^−1^). An obvious tendency is noted: with increasing current density, the discharge time decreases, since less time is needed to allow ion diffusion and redox reactions to occur at higher rates. However, the almost symmetrical triangular form at all the current densities depicts the great electrochemical reversibility and stability of the composite electrode. The fact that the discharge profile is retained even at higher currents also supports strong structural integrity and high charge transfer kinetics within the composite (MCM) electrode material, which supports its potential for high performance in supercapacitor applications [4,5,28]. In Figure 8a, the graph shows the transformation of the specific capacitance of the MCM composite at different current densities. A common decrease is observed when the current density increases, from a value of 1220 F g^−1^ at 1 A g^−1^ to 988 F g^−1^ at 2 A g^−1^, and further to 880 F g^−1^ at 3 A g^−1^ as the rate of charge–discharge is increased and the electrolyte ions are restricted in the diffusion velocity. Figure 8b shows the numerical values of the capacitance of the looped GCD curves and presents a quantitative evaluation of the electrochemical characteristics of the materials and composite. At a current density of 1 A g^−1^, the composite (MCM) displays a significantly high specific capacitance (1220 F g^−1^) that is more important than that of its constituent elements MoS_2_ (620 F g^−1^), CeO_2_ (300 F g^−1^), and MXene (290 F g^−1^) in panel (b). This improved composite (MCM) performance highlights the synergistic characteristics of its components: MoS_2_ provides a vast surface for ion adsorption, CeO_2_ provides redox-active sites, and MXene can offer high electronic conductivity and structural stability [25,36].The ternary composite (MCM) exhibited good cyclic stability with a capacitance value of about 90% of its original value after 5000 cycles as shown in Figure 9. The high retention indicates the structural stability and excellent electrochemical reversibility of the composite material. The very small capacitance loss (~10%) after this extensive cycling proves that the MCM composite is a good candidate as an electrode material for high-performance capacitors, especially for long-term durability and stability. Nevertheless, the composite does not lose much capacitance, meaning that the capacitance rate is very high and can be used in high-performance supercapacitors.

### 3.3. Electrochemical Impedance Spectroscopy (EIS)

**Figure 10:**
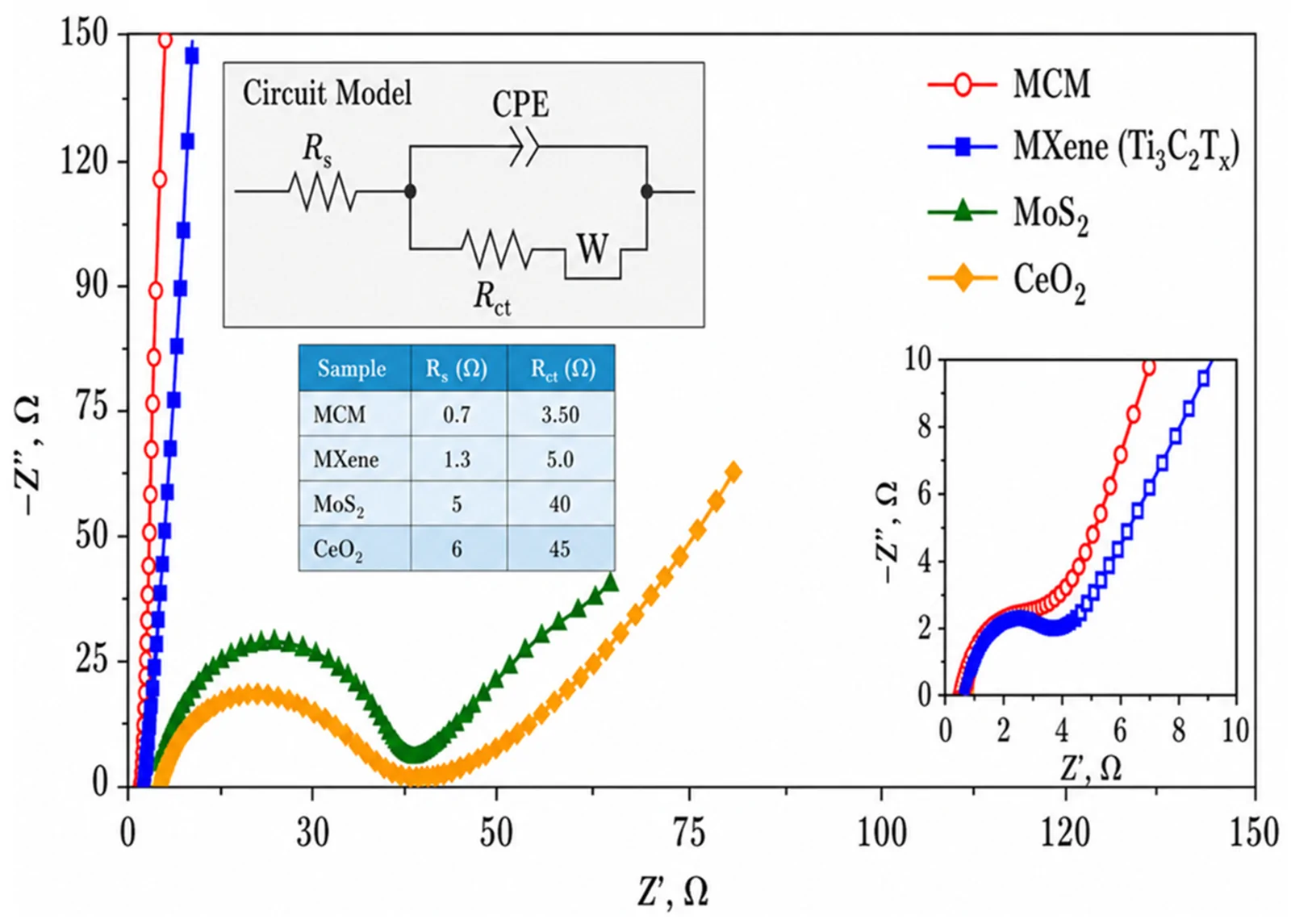
Electrochemical impedance spectroscopy (EIS) analysis of MXene, MoS_2_, CeO_2_, and the their composite (MCM).

To further elucidate the electrochemical kinetics and interfacial charge transfer behavior of the electrodes, electrochemical impedance spectroscopy (EIS) measurements shoewn in Figure 10 were conducted. The Nyquist plots were analyzed using the equivalent circuit, where CPE and W represent the solution resistance, charge transfer resistance, constant phase element, and Warburg diffusion impedance, respectively. Among all samples, the composite (MCM) exhibited the lowest (~0.8 Ω) and the smallest (~4 Ω) values, indicating excellent electrical conductivity and rapid interfacial charge transfer kinetics. MXene showed comparable impedance characteristics with a slightly higher (~5 Ω), consistent with its highly conductive two-dimensional layered structure [2,14]. CeO_2_, however, showed a larger semicircle and higher real impedance, indicating poorer electronic conductivity and low electrochemical activity, as expected from its CV behavior [21,22]. In comparison, MoS_2_ and CeO_2_ showed slower electrochemical reaction kinetics [39]. Furthermore, the low-frequency region of the composite (MCM) approached a nearly vertical line, suggesting faster ion diffusion and ideal capacitive behavior, whereas CeO_2_ exhibited the longest Warburg diffusion region, indicating greater diffusion limitations. These results confirm that the composite (MCM) possesses the lowest internal resistance, fastest electron transport, and most efficient ion diffusion, which are expected to translate into superior and long-term electrochemical performance.

## 4. Materials and Methods

### 4.1. Materials

The following chemicals were used in our experimental work: Ti_3_AlC_2_, HF, MoS_2_, and CeO_2_. These materials were purchased from Sigma-Aldrich.

### 4.2. Synthesis of Ti_3_C_2_T_x_ MXene

The HF etching process proved to be a very efficient way of obtaining Ti_3_C_2_T_x_ MXene. To start with, Ti_3_AlC_2_ (500 mg) was dissolved in a 15 mL solution of 10.5 mL of 48 percent concentrated HF and 5.5 mL of deionized water. In a magnetic hot plate, this mixture was put in a Polytetrafluoroethylene (Teflon) bar. Slowly but surely, Ti_3_AlC_2_ MAX phase was added to the solution at room temperature over a duration of 3 min, continuously stirring to avert any possible exothermic reaction. The Teflon bar was firmly capped, and the temperature was raised to 40 °C and continuous stirring was carried out over 18 h. The resulting solution was centrifuged at 5000 rpm at room temperature and washed 5 times with deionized water and 2 times with ethanol and water solution until the pH was more than 6, nearly 7. The respective supernatant was moved into a vacuum oven and dried at 70 °C overnight.

### 4.3. Composite Synthesis of Composite (MCM)

**Figure 11:**
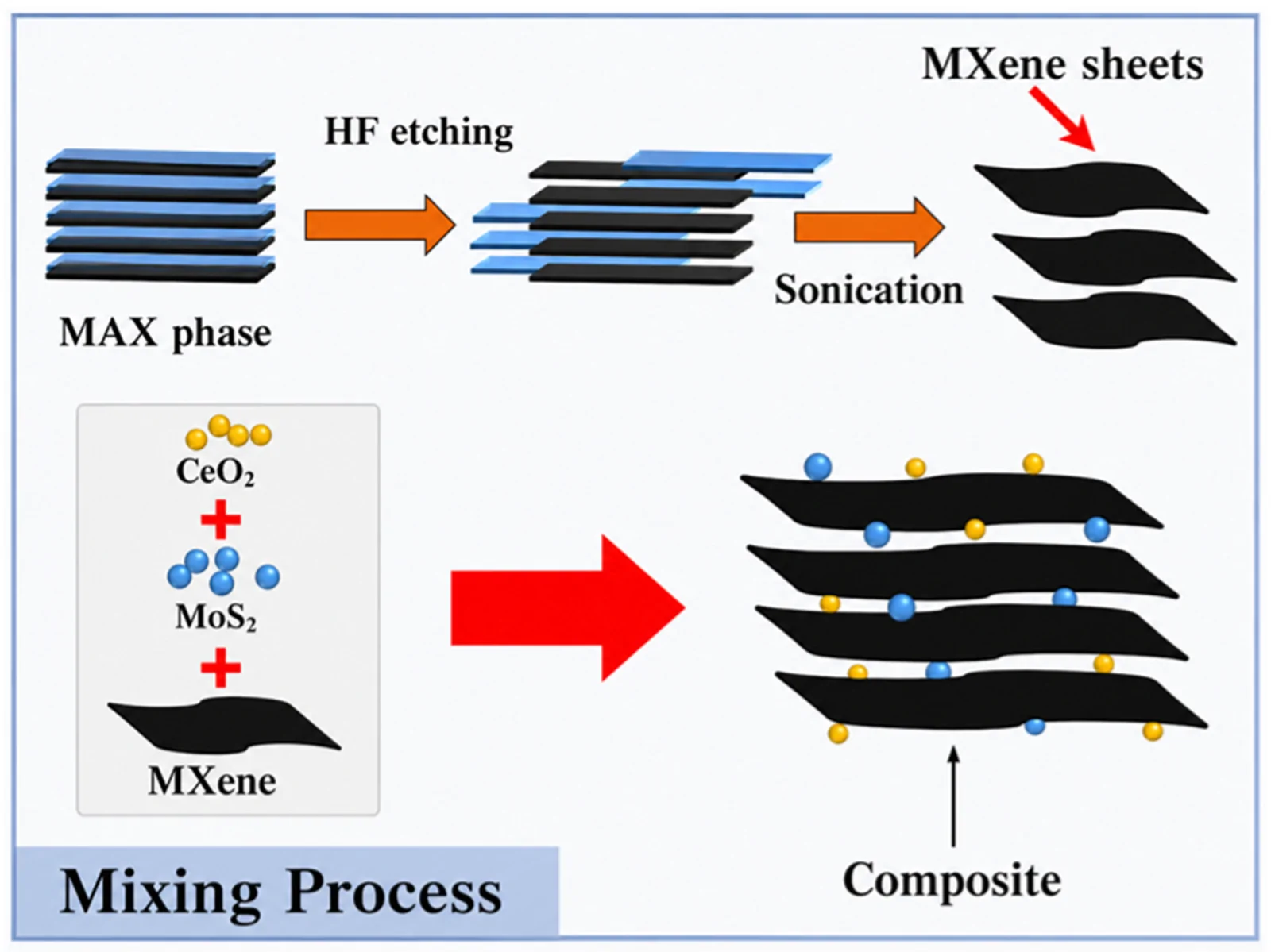
Schematic illustration of the synthesis process of the CeO_2_–MoS_2_@MXene composite. The orange arrows indicate the sequential processing steps, including HF etching and sonication, leading to the formation of exfoliated MXene sheets. The red arrow indicates the mixing and integration of CeO_2_, MoS_2_, and MXene to form the final composite. The red pointer arrow highlights the obtained MXene sheets, while the black arrow identifies the final composite structure.

Equal amounts of Ti_3_C_2_T_x_ (100 mg), MoS_2_ (100 mg), and CeO_2_ (1:1:1 by mass) were dispersed in separate glass beakers containing 20 mL of DI water each and stirred at 500 rpm for 4 h at room temperature. After stirring, all three solutions were combined into a single container and the mixture was stirred for an additional 50 min. Then, the mixed solution was transferred into a Teflon-lined autoclave. We set the autoclave temperature at 180 °C and maintained this temperature for 4 h. Finally, the product was recovered by centrifugation, and then washed 5 times with deionized water and once with ethanol. The final product was dried at 60 °C for 12 h under vacuum. During hydrothermal treatment, CeO_2_ and MoS_2_ particles were deposited on the surface and interlayer regions of the MXene sheets. The surface functional groups of MXene provided potential attachment sites for the other components. Simultaneously, hydrothermal pressure and temperature promoted interfacial contact and improved the integration of CeO_2_, MoS_2_, and MXene. This process produced an interconnected CeO_2_–MoS_2_@MXene hybrid structure while limiting particle agglomeration and the restacking of MXene sheets. The resulting composite material is now ready for further characterization and can be used in supercapacitor applications. The synthesis pathway of the CeO_2_-MoS_2_@MXene composite is illustrated in Figure 11.

### 4.4. Characterization

Using different characterization techniques, such as XRD, FTIR, Raman, BET, SEM, and EDX, the purchased and synthesized samples were characterized.

### 4.5. Electrode Fabrication and Electrochemical Measurement Configuration

The working electrodes were prepared using nickel foam (NF) as the current collector. Before coating, the nickel foam was cut into 1 × 1 cm pieces and rigorously cleaned using dilute HCL, deionized water, and ethanol to remove the surface oxides and other impurities. The homogeneous slurry was prepared by mixing the active material, polyvinylidene fluoride (PVDF) binder, and carbon black in a mass ratio of 9:0.5:0.5, respectively. The prepared slurry was uniformly coated onto a nickel foam (NF) substrate, maintaining an active-material mass loading of approximately 1.0–1.5 mg. The prepared electrodes were then dried overnight in a vacuum oven at 60 °C. All electrochemical measurements were performed using a GAMRY electrochemical workstation in a conventional three-electrode cell containing 3 M aqueous KOH electrolyte. Cyclic voltammetry (CV) measurements were conducted over a potential window of 0–0.6 V versus Ag/AgCl at scan rates of 10, 20, 30, 40, 50, 60, and 70 mV s^−1^. Galvanostatic charge–discharge (GCD) measurements were performed between 0 and 0.5 V versus Ag/AgCl at current densities of 1, 2, 3, 4, and 5 A g^−1^.

**Figure 12:**
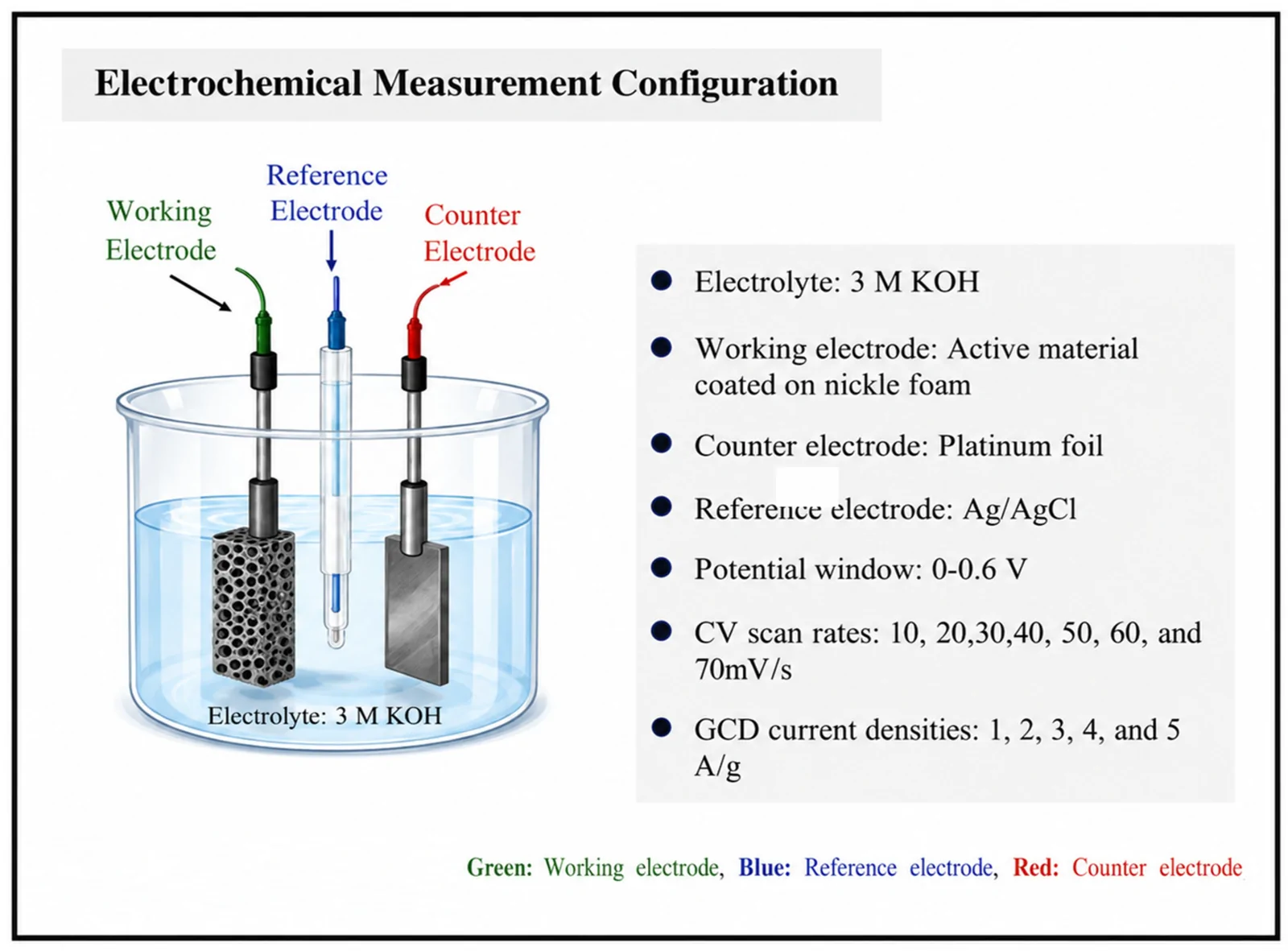
Electrochemical measurement configuration of the samples.

The specific capacitance (C_s_) was calculated from the GCD curves using the following equation:

C_s_ = IΔt/(mΔV)

where C_s_ is the specific capacitance (F g^−1^), I represents the discharge current (A), Δt is the discharge time (s), m denotes the mass of the active material (g), and ΔV is the effective discharge potential window (V) after excluding the IR drop. Electrochemical impedance spectroscopy (EIS) is reported in the frequency range of 100 kHz to 0.01 Hz. Details are given in Figure 12.

## 5. Theoretical Methodology

The plane wave Density Functional Theory framework calculation was performed using the VASP (Vienna ab initio simulation package) [40,41]. PBE-GGA is an exchange-correlation potential that is employed to assess the stability of structures [42]. Nonetheless, the electronic property calculations were done using the Heyd–Scuseria–Ernzerhof (HSE-06) functional because it gives more precise results than PBE-GGA [43]. The projector augmented wave (PAW) technique was used, and the cut-off energy was fixed to 530 eV [44,45]. Using the conjugate gradient technique, atomic positions and lattice parameters were fully relaxed until force and energy convergence values of 0.01eV/A and 1 × 10^−6^ eV/cell, respectively, were achieved. In the case of k-point sampling, we used the Monkhorst-Pack (MP) scheme with an MP grid [46,47], which has been proven to be accurate in earlier research [48]. Vacuum space (35A) was introduced to prevent contact between periodic images. Furthermore, the studied configurations were also determined incorporating van der Waals interactions, and dispersion corrections were provided using the DFT-D3 method [49].

### 5.1. Structural Stability

**Figure 13:**
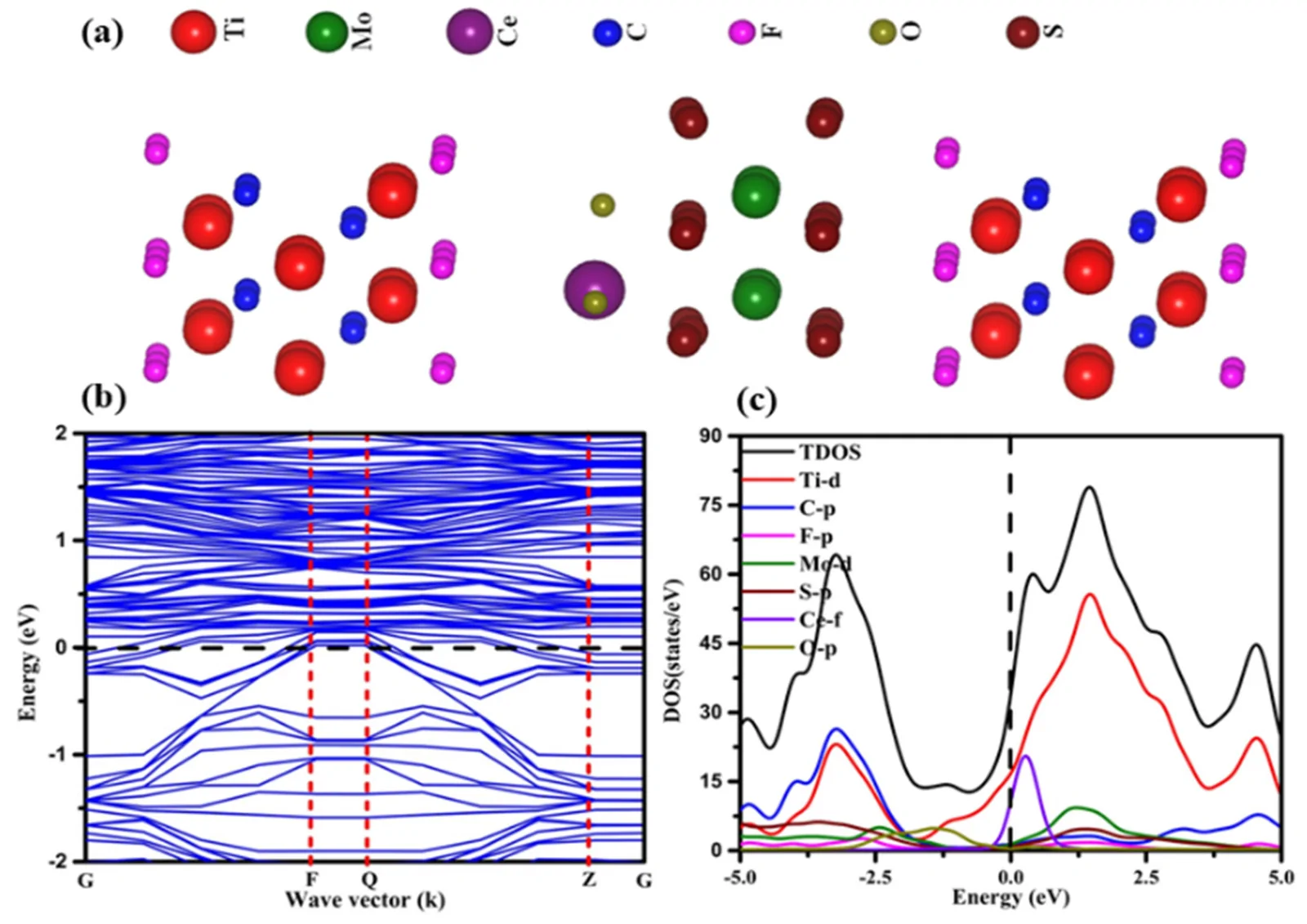
(**a**) Optimized structure of MoS_2_–CeO_2_ @MXene composite. (**b**) Band structure of MoS_2_–CeO_2_@ MXene composite. (**c**) TDOS and PDOS of MoS_2_–CeO_2_@MXene composite.

First, the Ti_3_C_2_F_2_ and MoS_2_ monolayers were fully relaxed. The unit cell of Ti_3_C_2_F_2_ is made up of two carbon (C) atoms, two fluorine (F) atoms, and three titanium (Ti) atoms. The calculated lattice constant of Ti_3_C_2_F_2_ was determined to be 3.31 Å, which is in good agreement with the previous result [50]. The important bond lengths, such as Ti-F and Ti-C, were recorded as 2.16 Å and 2.02 Å, respectively. The angles of bonding of F-Ti-F, Ti-C-Ti, and C-Ti-C were calculated to be 97.24°, 96.69°, and 90.19°, respectively. One Molybdenum atom and two sulfur atoms were contained in the MoS_2_ unit cell. Bond length (Mo-S) and bond angle (S-Mo-S) were also reported as 2.39 Å and 83.06 Å, respectively, which is also in agreement with previous reports. The lattice mismatch between the Ti_3_C_2_F_2_ and MoS_2_ layers is appropriate to model the hetero-trilayer structure. CeO_2_ nanoparticles are then deposited on the MoS_2_ layer surface after relaxation of the hetero-trilayer structure. The optimal structure is presented in Figure 13a. In all calculations, dispersion correction is done using the DFT D3 method [49], which has the Becke–Johnson damping function. Table 1 gives the parameters of the van der Waals force field. The binding energy (Eb) is a significant parameter in the stability analysis of a structure, and a stable structure is defined by a negative value of binding energy [51,52,53]. The binding energy of the structure studied can be calculated using the following formula [54].

(1) $$E_{b}={(E}_{total}-2E_{MXene}-E_{{MoS}_{2}}-E_{{CeO}_{2}})/N$$

Here, “$E_{total}$”, “$E_{MXene}$”, “$E_{{MoS}_{2}}$”, and “$E_{{CeO}_{2}}$” are the total energy of the MoS_2_–CeO_2_ @MXene composite, MXene monolayer, MoS_2_ monolayer, and CeO_2_ nanoparticle, respectively. In this case, N indicates the sum of the number of atoms of MoS_2_-CeO_2_ in the composite of MoS_2_-CeO_2_ and MXene. The calculated binding energies of the MoS_2_-CeO_2_ @MXene composite are negative, which means that all the structures are energetically favorable and stable [50,51,52,53,54]. The MoS_2_-CeO_2_ @MXene composite has a binding energy per atom of −0.091 eV.

### 5.2. Electronic Properties

The DFT results indicate significant interfacial charge redistribution and electronic coupling among the MoS_2_, CeO_2_, and MXene components, suggesting enhanced charge transfer kinetics across the heterointerface. Such electronic interactions are consistent with previous studies demonstrating that oxygen vacancy-induced electronic-state modulation and orbital hybridization can facilitate electron transport, increase electrochemically active sites, and consequently improve charge storage performance [27,28].

Band structure and density of states (TDOS and PDOS) are calculated to explain the electronic properties of the MoS_2_–CeO_2_ @MXene composite using the Heyd–Scuseria–Ernzerhof (HSE-06) functional. HSE-06 gives better results for transition atoms compared to PBE-GGA. Figure 13b represents the band structure plot of the MoS_2_–CeO_2_ @MXene composite. The black horizontal dashed line represents the Fermi level at 0eV and divides the graph into two regions. The regions above and below the Fermi level are known as the conduction and valence bands, respectively. The calculation is being carried out along several high-symmetry points (G→F→Q→Z→G). In Figure 13b, it is also seen that the conduction and valence bands are overlapping at the Fermi level; hence, no bandgap is observed. Hence, the MoS_2_–CeO_2_@MXene composite is said to be metallic.

Figure 13c exhibits the total and partial density of states (TDOS and PDOS) of the MoS_2_–CeO_2_@MXene composite. The vertical dashed line represents the Fermi level and divides the graph into two regions (valence and conduction band). In this depiction, it is evident that there is no gap between the valence and conduction bands, confirming the metallic nature of the composite. Thus, DOS results also match the band structure. This feature is primarily attributed to the MXene phase, which provides a conductive backbone for electron transport. The PDOS analysis shows clear contributions of Ti-d, Ce-f, Mo-d, S-p, C-p, and O-p states. These findings indicate that in the conduction band, the orbital of the Ti atom makes its contribution to the conduction band. In the valence band, its TDOS is caused by the equal contribution of the orbital of C and Ti, that is, the C-p orbital and the Ti-d orbital. Ce-f orbitals created a peak close to the Fermi level (EF) that increased the conductivity of electrons. The MoS_2_ exhibits an EKG derived mainly by Mo-d and S-p orbitals. The presence of a single peak of Ce-f bands indicates localized electronic states, which are useful for enhancing dielectric and redox activity. Such alignment facilitates effective charge separation, inhibits recombination, and increases ion accessibility. The structural intercalation and electronic hybridization enable the composite to provide high-rate electrochemical activity, making it a good candidate for high-rate energy storage applications.

## 6. Conclusions

In this work, an MoS_2_-CeO_2_@MXene (MCM) composite was successfully synthesized and evaluated as a high-performance electrode material for energy storage applications such as supercapacitors. Structural and spectroscopic analyses, including XRD, FTIR, Raman spectroscopy, SEM, and EDX analysis, confirmed the successful synthesis of the composite (MCM) and the effective integration of its constituent components. The electrochemical measurements were systematically investigated using cyclic voltammetry (CV), galvanostatic charge–discharge (GCD), electrochemical impedance spectroscopy (EIS), and cycling stability measurements in a three-electrode system. The electrode of the synthesized composite delivered a higher specific capacitance of 1220 F g^−1^ than the individual components and exhibited excellent cycling durability, with 90% capacitance retention after 5000 charge–discharge cycles. The electrochemical performance can be attributed to the synergistic interaction among MoS_2_, CeO_2_, and MXene, which provides abundant electroactive sites, improved charge transfer kinetics, and efficient ion diffusion pathways. Furthermore, the DFT results agree with the experimental results, revealing favorable electronic interactions within the hybrid composite and supporting the observed enhancement in charge storage performance. These results demonstrate that the MCM composite is a promising candidate for the development of next-generation high-performance supercapacitor electrodes.

## Figures and Tables

**Table 1:** Parameters of the vdW force field.

Atoms	C_6_	R_o_
	Jnm^6^/mol	Å
Ti	10.8	1.562
C	1.75	1.452
F	0.75	1.287
Mo	24.67	1.639
S	5.57	1.683
Ce	140.68	1.753
O	0.70	1.342

## Data Availability

The data will be available on request.
